# Genetic Code Expansion for Mechanistic Studies in Ion Channels: An (Un)natural Union of Chemistry and Biology

**DOI:** 10.1021/acs.chemrev.4c00306

**Published:** 2024-08-29

**Authors:** Daniel T. Infield, Miranda E. Schene, Jason D. Galpin, Christopher A. Ahern

**Affiliations:** Department of Molecular Physiology and Biophysics, University of Iowa, Iowa City, Iowa 52242, United States

## Abstract

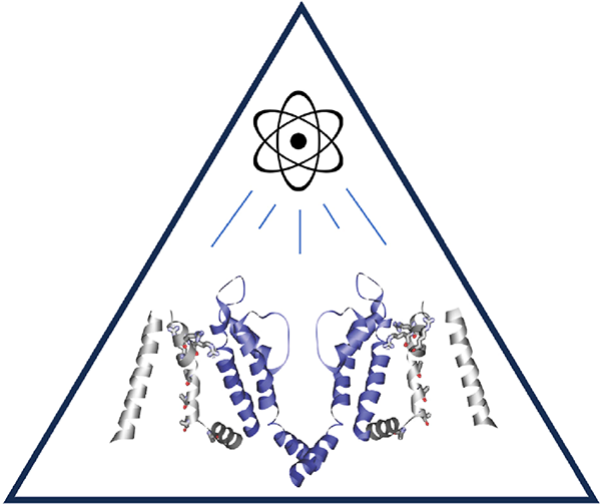

Ion channels play central roles in biology and human health by catalyzing the transmembrane flow of electrical charge. These proteins are ideal targets for genetic code expansion (GCE) methods because it is feasible to measure ion channel activity from miniscule amounts of protein and to analyze the resulting data via rigorous, established biophysical methods. In an ideal scenario, the encoding of synthetic, noncanonical amino acids via GCE allows the experimenter to ask questions inaccessible to traditional methods. For this reason, GCE has been successfully applied to a variety of ligand- and voltage-gated channels wherein extensive structural, functional, and pharmacological data exist. Here, we provide a comprehensive summary of GCE as applied to ion channels. We begin with an overview of the methods used to encode noncanonical amino acids in channels and then describe mechanistic studies wherein GCE was used for photochemistry (cross-linking; caged amino acids) and atomic mutagenesis (isosteric manipulation of charge and aromaticity; backbone mutation). Lastly, we cover recent advances in the encoding of fluorescent amino acids for the real-time study of protein conformational dynamics.

## 1 Introduction

**Figure 1:**
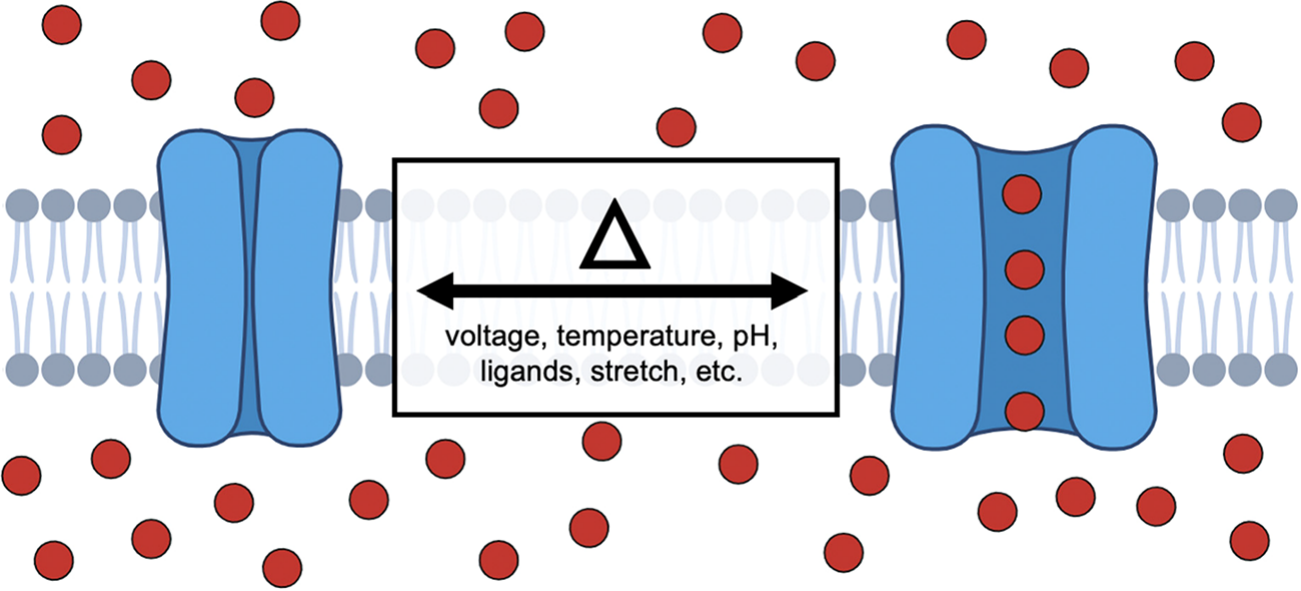
Schematic view of an ion channel as modulated by external stimuli.

Ion channels are essential for cellular life. In their simplest form, these transmembrane proteins enable the passage of charged ions through the otherwise impenetrable hydrophobic core of a phospholipid bilayer (Figure 1). This feat is achieved by creating a water-filled proteinaceous pathway that mimics the liquid environment of the ion in free solution. Ion channels have become so adept at this role that they can improve membrane transport rates by over 10 million-fold over bulk diffusion.^1^ In doing so, ion channels have evolved to enable ion transport and biological communication from bacteria to humans.^2,3^

The pore-domain of the channel can be coupled with a modulatory domain that serves to relay environmental information to “gate” ionic conductance.^4^ In the well-studied example of electrically excitable cells of nervous and muscle tissues, voltage-gated cation channels sense and respond to millisecond changes in transmembrane voltage by allowing the selective conductance of sodium, calcium, or potassium ions.^5^ This exquisite control of protein conformation is achieved through voltage-driven conformational changes in voltage-sensing domains that are relayed to the pore-domain.^6^ The interplay between these channels gives rise to rapid (millisecond) and reversible deflections in membrane voltage known as action potentials, the quanta of information in excitable cells that support thought, perception, sensation, and motility.

In postsynaptic ligand-gated ion channels, an exofacial ligand-binding pocket rapidly transduces neurotransmitter binding to channel activity.^7,8^ Such ligand-gated channels are essential for neurological, sensory, cardiac, and skeletomuscular signaling. There are also ion channels that have evolved to sense and respond to changes in temperature.^9^ These are found in sensory organs and within the autonomic nervous system, where they integrate and regulate body temperature. These same channels are the targets of noxious agents such as capsaicin, mustard oil, camphor, and menthol which trick our senses to feel heat or cold from chemical stimulation.^10^

Additional ion channel proteins are capable of sensing and responding to cellular swelling and pressure through purpose-built mechano-sensation domains.^11,12^ In so doing, these channels convert stretch to electrical signals during touch,^12^ proprioception,^13^ fat metabolism,^14,15^ and glucose homeostasis.^11^ Other ion channels mediate homeostatic processes, such as osmotic regulation of epithelial cells through chloride conductance (e.g., CFTR^16^ and CaCC^17^). The intense study of ion channels stems from their prominent roles in health and disease; loss-of-function and gain-of-function genetic mutations underlie dozens of ion channelopathies^18^ and the drugging of normal and mutant channels has been leveraged to treat various conditions including cardiac arrythmia^19^ and postoperative pain.^20^

Ion channels are particularly apt for the application of genetic code expansion (GCE) and therefore have been interrogated in numerous chemical biology studies with the encoding of noncanonical amino and α-hydroxy acids.^21^ This appropriateness is intrinsic to the function of the ion channel. The transmembrane flux of a charged ion produces a measurable electrical current: a single channel can conduct up to 1 million ions per second across a membrane bilayer, representing a trillionth of an ampere or picoamp (pA). For almost 100 years, methodologies known collectively as “electrophysiological” techniques have allowed the study of these small ionic currents through amplification.^22^ The ability to accurately measure the activity of miniscule amounts of ion channel proteins—even single channels^23^—is advantageous when combined with the sometimes low-yield chemical biological approaches for the encoding of noncanonical amino acids. This real-time, direct observation of ion channel function also lends itself to established biophysical approaches and methods of analysis.^24^

## 2 Methods for Encoding Noncanonical Amino Acids

**Figure 2:**
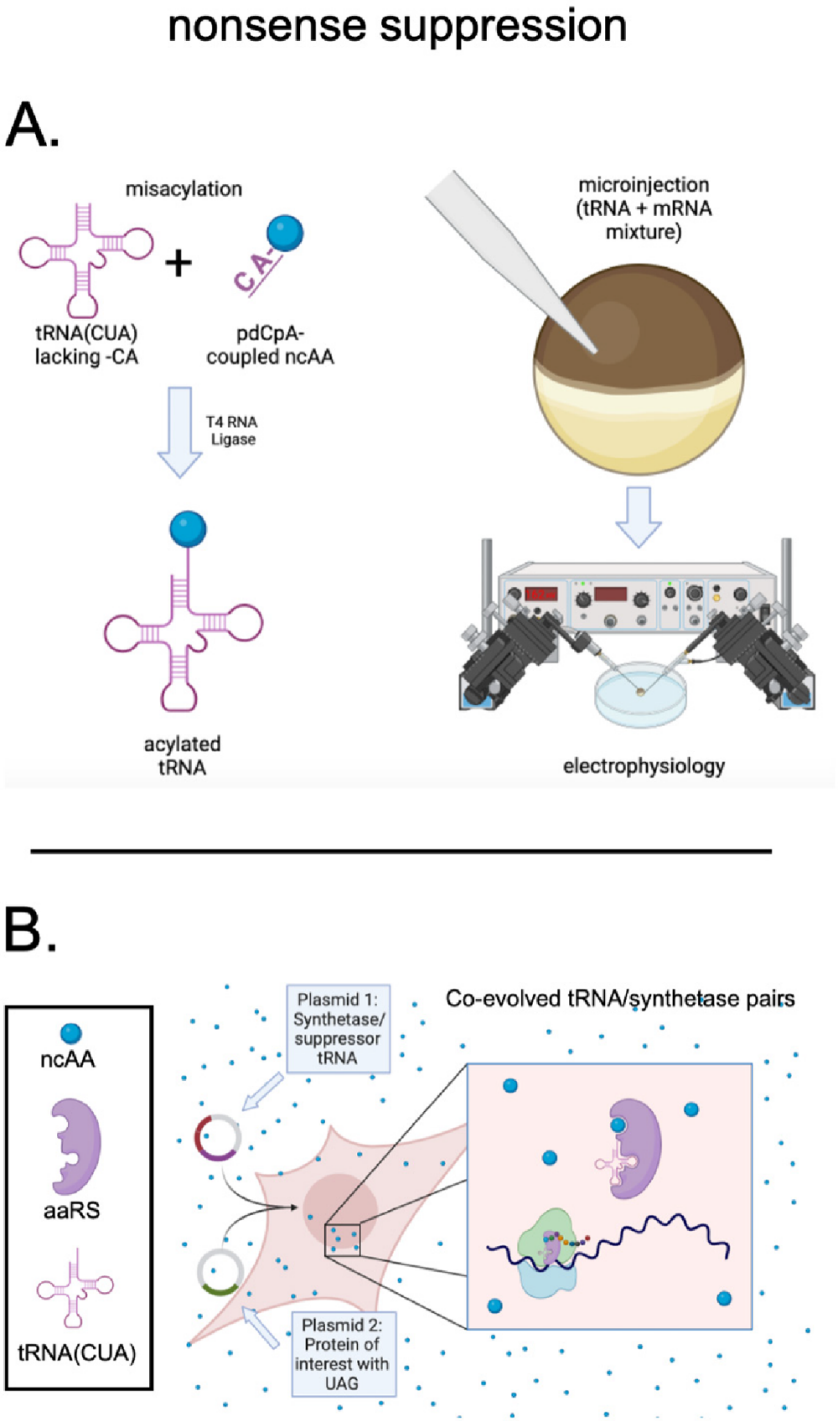
Nonsense suppression for genetic code expansion. A. Chemical misacylation of stop codon suppressor tRNA, and subsequent coinjection with mRNA. B. Cotransfection of plasmids encoding a tRNA/synthetase pair specific for an ncAA, and the protein of interest with introduced TAG (mRNA codon UAG), in the presence of ncAA.

**Figure 3:**
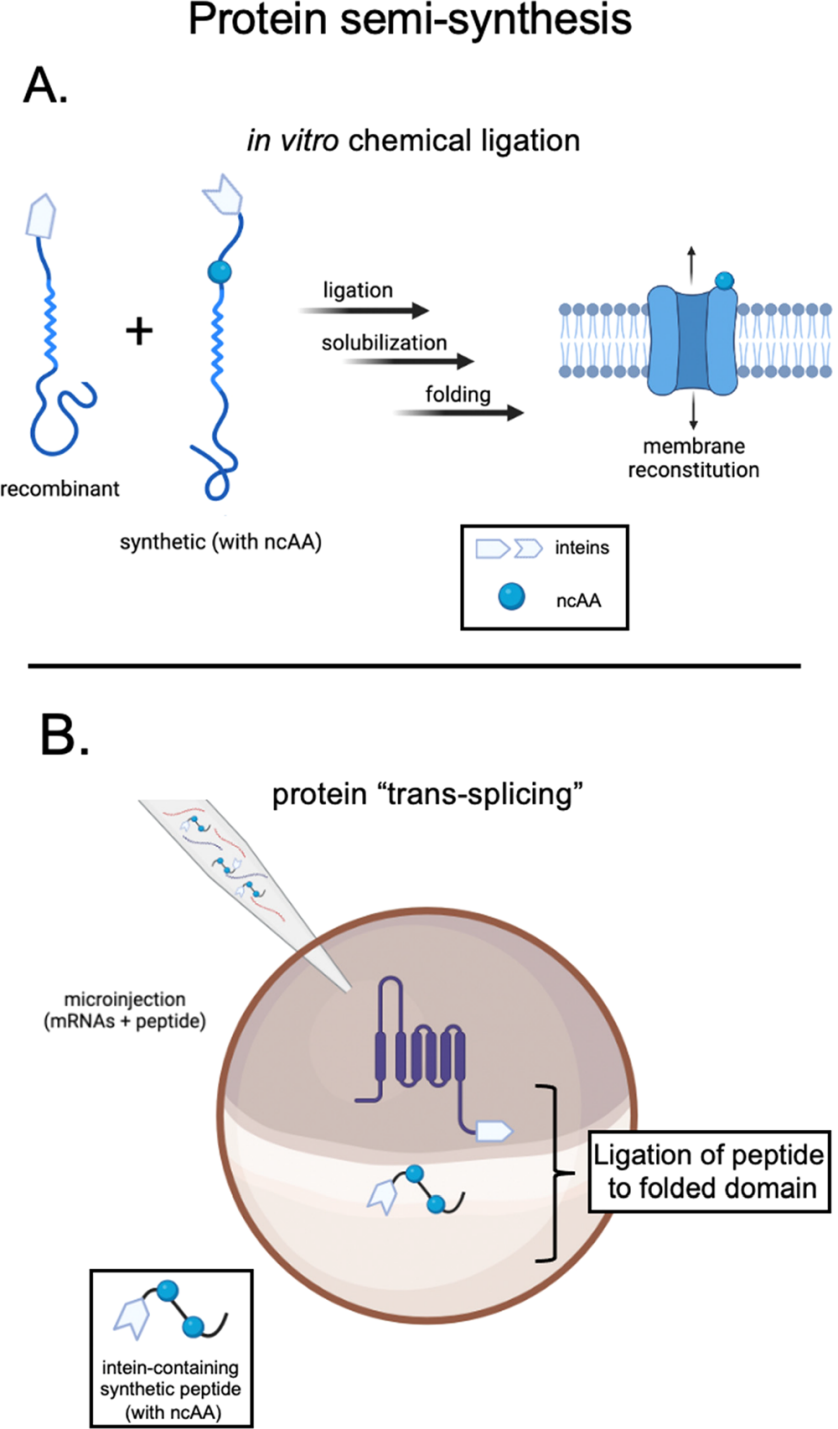
Methods for production of engineered, semisynthetic ion channels. A. *In vitro* route requiring folding and subsequent reconstitution in artificial liposomes. B. Protein trans-splicing involves coinjection of recombinant and synthetic components, which are then ligated together in-cell via inteins.

Within the scope of this review, we focus on the chemical biology methods used to encode non-natural (synthetic) amino acids into ion channels, rather than chemical post-translational modification of natural residues (e.g., cysteine modification^25^ and amine coupling^26^). The relevant GCE methods can be broadly divided into those based on nonsense suppression (Figure 2),^27^ or protein semisynthesis^28^ (Figure 3).

In the former, a stop codon, most commonly the amber stop codon TAG- is introduced in the DNA, and thus RNA (UAG) at the intended position of encoding within the protein of interest. During translation, the corresponding mRNA UAG triplet is recognized by a codelivered suppressor tRNA (for UAG, the anticodon CUA) that is acylated with the noncanonical amino acid. The orthogonal suppressor tRNA used is often a natural amber codon reader, of which the glutamine tRNA (*THG73*) from the unicellular eukaryote *Tetrahymena thermophil*a^29,30^ and the pyrrolysine tRNA (*PylT*) from methanogenic archaea^31−33^ are prominent examples. Alternatively, the anticodon of a natural tRNA may be edited to recognize the amber stop codon.^34^ Some suppressor tRNAs, including *PylT*, have proven amenable to recognize other triplet stop codons (UAA, UGA)^35^ or quadruplet^36^ codons; this enables the potential for site-specific encoding of multiple noncanonical amino acids within a single protein. In a cellular context, it is important that the tRNA be orthogonal in the system of application, meaning it is competent for translation by the ribosome, but poorly- or unrecognized by endogenous aminoacyl tRNA synthetases, which could lead to reacylation and spurious incorporation of natural amino acids instead of the synthetic amino acid of interest.^37^

In the chemical misacylation (Figure 2A) strategy, the protected synthetic amino acid is coupled to a dinucleotide CA-analog via an activated cyanomethyl ester^38^ and then enzymatically ligated to a folded tRNA precursor lacking the terminal C and A nucleotides. The side chain functional group and α amine may be protected with entities such as the 2-nitroveratryl group^29^ for photodeprotection, pentenone amide^39,40^ for mild iodine deprotection, or t-butyl carbonyl^41^ for removal with TFA. The latter group is taken off prior to the enzymatic ligation step owing to tRNA sensitivity. Once the aminoacylated tRNA is isolated as a dry pellet it is stable at −80 °C, but upon dissolution and photo/chemical deprotection it is sensitive to hydrolysis at alkaline pH and elevated temperatures.^42^ This irreversible hydrolysis can be a significant factor in choosing which types of amino acid chemistries to encode. tRNA featuring amino acids with neutral side chains or α-hydroxy analogues have much greater stability in a neutral pH aqueous buffer, possibly owing to the lack of charge density stabilizing the approach of hydrolytic water.^43^

The chemically misacylated tRNA is delivered to the cell cytosol by microinjection (Figure 2A) or, in cell free experiments, simply added to the reaction. A major advantage of the chemical misacylation method is its flexibility: theoretically, any amino acid that can be coupled to the pdCpA dinucleotide can be used to acylate an already proven suppressor tRNA, thus limiting encoding to compatibility with ribosomal translation. Limitations of the system relate chiefly to the requirement for microinjection and the fact that the acylated tRNA is often unstable, particularly in the absence of translation elongation factor(s),^42^ and is nonrenewable in cells. These issues limit the types of amino acids utilized in practice and the efficiency of incorporation, particularly over long incubation periods. This may be a serious issue if very high expression of the protein of interest is required for measurable signals; in this case, success has been achieved by performing additional tRNA injections in the days after the initial mRNA/tRNA injection (tRNA “boosting”).^44^

A powerful alternative to the chemical misacylation method is the identification of coevolved tRNA/synthetase pairs for the incorporation of noncanonical amino acids into ion channels (Figure 2B). The synthetase method enjoys compatibility with scalable recombinant protein expression systems that is unique in GCE, owing to the presence of the coexpressed ncAA-encoding aminoacyl-tRNA synthetase in the cell. That is, unlike chemically acylated tRNA methods described previously, the aminoacyl-tRNA synthetase continuously reacylates the tRNA in the cell during protein production. Technical hurdles are also minimized, as many popular ncAAs for ion channel study are commercially available and therefore the method is accessible to essentially any group with expertise in cell culture and molecular biology. Note that in this system, orthogonality must extend beyond the tRNA to include the aminoacyl-tRNA synthetase and the ncAA being used.

An early effort to create orthogonal synthetase systems was undertaken using the glutaminyl-tRNA synthetase (Gln *R*S) from *E. coli*, which underwent extensive mutation and modification to make the system orthogonal and exclusively recognize amber stops.^45^ This system was used in prokaryotic systems, and it still mildly recognized the endogenous Gln-tRNA, but it nonetheless served as a major conceptual advance. Cross-kingdom strategies and applications (e.g., using a prokaryotic system in eukaryotes) were pursued to enhance selectivity and prevent endogenous recognition. For example, subsequent work adapted the tyrosyl-tRNA synthetase system from *E. coli* for use in a eukaryotic system, specifically *S. cerevisiae*.^46^ Multiple independent systems of tRNA/synthetase pairs have been developed and are in use, and the pyrrolysyl–tRNA system from the archaea *Methanosarcina barkeri* and *Methanosarcina mazei* has emerged as particularly useful in both bacterial and mammalian systems.^47−50^ This archaeal system already has a tRNA that recognizes the TAG codon to encode the “22^nd^ amino acid” pyrrolysine, and the synthetase is particularly permissive, allowing active site mutagenesis to significantly expand its amino acid recognition potential.^32,51^ Libraries of pyrrolysine systems have since been developed based on this system, and it is a widely used tool for ncAA encoding, particularly in mammalian cells.^52−54^ Efforts to identify improved systems with higher efficiency encoding and with greater diversity in the type of amino acids encoded are ongoing.

A family of methods that are overall independent from nonsense suppression for site-specific installation of noncanonical amino acids in ion channels involves the synthesis of a fragment (peptide) of the protein of interest containing the noncanonical amino acid and then subsequent ligation of this peptide to those that cover the rest of the channel sequence. (Figure 3). Such a strategy is considered “semi-synthetic” when the peptide segments that do not contain the noncanonical amino acid are generated via recombinant DNA technology.

A variety of routes are taken to combine these peptides into a functional engineered ion channel. In a fully *in vitro* embodiment of the method, these peptides are spliced together using native chemical ligation with inteins,^55^ after which the full-length channel polypeptide is folded and reconstituted into a lipid bilayer (Figure 3A). This method carries the advantage of chemical-synthetic control and can result in near-quantitative production of final product,^55^ but it is limited to those proteins of interest that are amenable to refolding and reconstitution. In an alternative and complementary method, called “tandem protein trans-splicing,” a synthetic peptide containing the noncanonical amino acid(s) is coinjected alongside the mRNA encoding the natural domains of the channel into a large cell (such as a *Xenopus* oocyte).^56,57^ The semisynthetic ion channel is assembled via *in vivo* intein ligation of the cotranslationally folded, recombinant domains to the coinjected synthetic peptide. This method is highly desirable in scenarios wherein the protein target must be expressed in a eukaryotic cell and the noncanonical amino acids of interest are refractory to nonsense suppression.^58^ The major limitations are the low efficiency of ligation *in vivo*,^56^ and restriction to locations and regions on proteins that are amenable to splicing postfolding (unstructured or solvent accessible regions of domains, N or C termini, etc.^59^).

## 3 Photochemistry in Ion Channels

### 3.1 Caged Amino Acids

**Figure 4:**
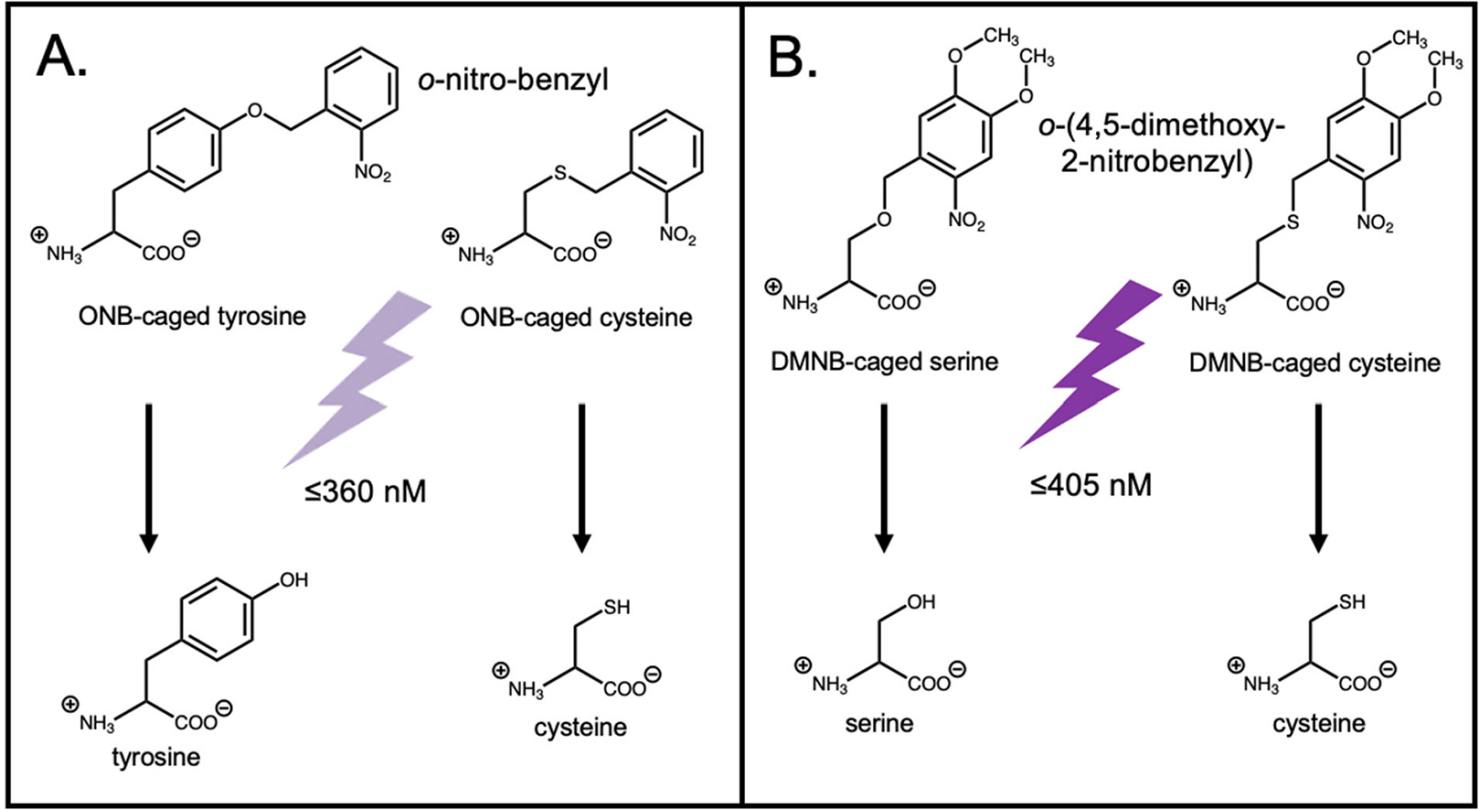
Photocaged unnatural amino acids. A. ONB-caged amino acids require potentially harmful ≤360 nm wavelength UV light. B. DMNB-caged amino acid absorption extends to borderline-visible (≤405 nm) light.

Photochemistry is a powerful and widely adaptable tool for the control and quantitative interrogation of biological processes.^60^ Within this broad field is a set of methods concerning the chemical “photocaging” of biomolecules in such a way that their active form is revealed by a pulse of high energy light. In the context of GCE for ion channels, the site-specific installation of caged amino acids has been applied to control protein function and conformation in membrane proteins. Pioneering reports from the Lester and Dougherty groups utilized the flexible chemical misacylation approach^29^ to encode o-nitrobenzyl caged tyrosine^61^ and cysteine^62^ (Figure 4A) within ligand-gated ion channels (e.g., nAChR) in whole (injected) *Xenopus* oocytes. The effects of photodecaging in these early studies resulted in a variety of complex phenotypes.^61^ This outcome was possibly due to 1) optical limitations in UV treatment of the large, opaque *Xenopus* oocyte and/or 2) an apparent effect of caging the tyrosine in forcing the channel into nonphysiological conformation(s) before treatment with light.^61^

A subsequent step forward was reported by the Schultz group^34^ in the identification of a tRNA and synthetase (RS) pair specific for a photocaged cysteine analog. The pair is derived from the *E. coli* leuRS/tRNA^LEU^ system for amber suppression in yeast,^34^ which is also orthogonal in other eukaryotic systems, including mammalian cell lines.^63^ The synthetase/tRNA pair thus enabled scalable expression of the photocaged engineered proteins within cell types that are far more amenable to optical methods than the *Xenopus* oocyte. This system subsequently yielded additional synthetases with active sites specific for related caged amino acids, including DNMB caged serine.^64^ Of note, unlike the o-nitrobenzyl group, the DNMB cage has absorption in the visible spectrum that is sufficient to allow rapid decaging with purple and blue light and thus avoiding the phototoxic effects of UV-A and UV-B radiation (Figure 4B).^64^ More recently, related synthetase/tRNA pairs and chemical misacylation schemes have led to the encoding of caged lysine,^65^ caged histidine,^66,67^ and caged glutamate^68^ via nonsense suppression, although to our knowledge, these have not yet been utilized in mechanistic ion channel studies.

The original stated purpose and most common application of these tools is as biological “photo-switches.” For example, the caged serine synthetase has been used to encode an isosteric DMNB-caged cysteine amino acid within the pore domain of K_ir_ channels.^69^ This approach produces an engineered, blocked K_ir_ channel which can be rendered conductive by photoremoval of the DMB side-chain. This approach allows for light-switched hyperpolarization in HEK cells and neurons.^69,70^ Along the same lines, caging of amino acids within the active sites of enzymes has been used to precisely control their activity (and related biological pathways) with brief flashes of light.^65^ Finally, key regulatory phospho-serine residues in proteins have been caged, with pulses of light enabling *in vivo* phosphorylation and relevant downstream signaling.^64^

Recently, we leveraged the encoding of DNMB-caged serine for a different purpose: to dissect the structural and functional roles of individual candidate phospho-serine residues in the complex and multivalent protein PKA regulation of the CFTR chloride channel.^71^ Several serine residues within the so-called regulatory “R” domain of CFTR have been implicated in channel activation (or inhibition) on the basis of historical studies using conventional mutagenesis,^72−74^ but their individual functional roles and potential coupling has been unclear. To approach this problem, we encoded caged serine into the R domain within 5 different PKA consensus sites in CFTR expressed in HEK cells and then interrogated the effect of their decaging (and subsequent phosphorylation) on channel function (real time–single cell electrophysiology) and biochemical state (phospho-specific antibodies).^75^ Using these methods in tandem allowed the demonstration of strong regulatory and functional coupling between two key stimulatory sites in the back half of the CFTR R domain, S795 and S813.

Within this study, to better understand the structural relevance of the DNMB photocage, we directly measured the affinity of the catalytic unit of PKA for recognition site variants. Importantly (if unsurprisingly), we found that the cage is an extremely strong deterrent to PKA binding as compared to serine, and even alanine mutation.^75^ It has been recently shown that simple binding of the catalytic domain of PKA (PKAc) transiently activates CFTR *in vitro*(76) and that this mechanism (functional effect of binding) may be generalizable.^77^ This general mechanism is likely relevant to other phosphoregulated membrane proteins, although the *in vivo* relevance is not yet fully understood. There is potential to use caged serine in the future to better understand, for specific sites, whether *bona fide* serine phosphorylation rather than simple kinase binding (which cannot be distinguished with alanine mutation) is critical for regulation.

### 3.2 Cross-Linking Amino Acids

A related class of encodable noncanonical amino acids are photoactivated cross-linkers. These are a class of noncanonical amino acids that are part of the broader class of photoactivated amino acids, which have side chains that change conformation or properties in response to light. These cross-linking noncanonical amino acids are generally stable under ambient temperature and light, but when directly irradiated, form reactive species that readily form covalent bonds with nearby molecules.^78^ The radius of these covalent bonds is limited to a few Å around the residue, and the formation of these bonds is largely nonspecific, meaning that they can form bonds with any common functional group in the surrounding region after photoactivation.^79^ Two of the most common cross-linkers used in the study of protein structure and function are benzoyl-phenylalanine (Bpa) and p-azido-phenylalanine (AzF). Both are unnatural amino acids with benzene rings in their side chains, and they are often encoded in place of aromatic residues, which may minimize the potential functional consequences of ncAA encoding (before photoactivation). However, mutating aromatic residues to these ncAAs can have a range of functional consequences depending on the site. For example, neither AzF nor Bpa had functional consequences when incorporated in a site in the GluN2A channel, but when encoded in GluN2B, the channel activity was significantly altered.^80^ Therefore, the site of encoding must be chosen and analyzed carefully, to determine whether any changes in structure or function are due solely to the chemical properties of the cross-linker.

Photoactivated cross-linkers can be used to investigate protein–protein binding, either within subunits or between different soluble and/or membrane proteins. Photoactive cross-linkers have an advantage over other binding analysis methods in that they form covalent bonds, allowing copurification of bound proteins that may have only weak interactions and would otherwise not remain bound after cell lysis. For example, in this way the soluble protein PGRMC1 was found to bind to the T-cell membrane protein PD-L1 and regulate its function.^81^ Because the extraction of membrane proteins from mammalian cells requires relatively harsh lysis conditions, the interaction between these proteins would fall apart and not be identifiable in downstream analyses. The UV-activated cross-linker AzF was encoded into PD-L1 at the putative binding site, and the living cells were treated with UV radiation, which covalently captured PD-L1 and PGRMC1. In addition, due to the nature of the photoactivation, bonds will only form within a few Å of the ncAA, meaning that only closely bound proteins will be captured (thus reducing the effective appearance of nonspecific interactions).

In ion channels, AzF has been encoded into the acidic pocket of the ASIC1a acid-sensitive channel to investigate dynorphin binding.^82^ This study confirmed the importance of the binding pocket to the ASIC1a function. In another channel, Bpa has been used to investigate the subunits that form the Kv7.1 voltage-gated potassium channel.^83^ Kv7.1 is made of two protein components: 4 KCNQ1 subunits and between 2 and 4 KCNE1 subunits. Bpa was encoded in KCNE1, and when coexpressed with KCNQ1, a UV pulse resulted in a loss of current. The authors used this approach to determine that when 2 KCNE1 subunits were bound to 4 KCNQ1 subunits, additional KCNE1 subunits could bind, indicating that KCNE1 binding is not cooperative. Bpa has also been used to investigate heterodimers of ionotropic glutamate receptors.^84^ In particular, the authors used AzF to show that determinants of interaction of GluN2A and GluN2B with the GluN1 subunits differed. They encoded AzF in a particular location of GluN1 and exposed it to UV pulses when expressed with either Glu2A or Glu2B in heterodimers. They found that the GluN1/GluN2B heterodimer underwent irreversible channel deactivation but the GluN1/GluN2A heterodimer did not, indicating that these two different subunits have different binding modalities in heterodimers.

Photoactivated cross-linkers have also been used to investigate ion channel conformational states. Channels and transporters are highly motile proteins and change states rapidly and in response to a variety of cellular stimuli. Ion channels can exist in multiple open states (full versus subconductance) and multiple closed states (resting/deactivated, inactivated, desensitized). The differences between conformational structures in these states are very important to understanding channel physiology as well as for drug development. UV-activated cross-linkers have been used previously in ion channels to study conformation, via the use of soluble cross-linkers that bound to introduced cysteines.^85^ The use of photoactivated ncAAs has the advantage of being flexible as AzF or Bpa may be encoded in any desired area of the channel. Due to the property of covalently binding functional groups within a few Å of itself in a controllable manner, the cross-linker can be activated when the cell membrane is at a given voltage or in a specific state. If the ncAA cross-linker is in an appropriate position, the cross-linking reaction can immobilize, or “lock,” the channel in a given state. Depending on the specific question being asked, this can be a very useful tool. Questions regarding the previously mentioned ASIC1a channel were addressed in this manner.^86^ AzF and Bpa were encoded into a region of the channel known as the “acidic pocket,” and were cross-linked to immobilize it. By doing so, the researchers selectively destabilized the open state structure. AzF was used in a somewhat different method to investigate state-dependent structure in glutamate receptors.^87^ In this investigation, AzF was encoded in different regions of the channel, and then UV was applied in different states of the channel, either activated or deactivated. UV cross-linking resulted in a loss of channel function, which indicated AzF was located close to another functional group in that state. For some sites, UV cross-linking was achieved in one state but not the other, suggesting conformational changes in these regions. This, in conjunction with structural studies, provided Å-level resolution of amino acid position changes between states. The utility of photo-cross-linkers in glutamate receptors has been examined in further detail and refined in both AMPA and NMDA receptors.^88−90^ A similar approach has been used assess conformational changes in hERG channels bearing an encoded Bpa.^91^ The data here suggest that photochemical cross-linking reduces mobility in the cytoplasmic “PAS” domain, and this reduction may result in the stabilization of the closed state relative to the open state of the channel. Similarly, Goodchild and Ahern used encoded Bpa to study the relative motion of the putative voltage-gated sodium channel “inactivation gate” formed by the cytoplasmic linker between channel domains III and IV.^92^ Here, cardiac sodium channels were expressed in HEK 293t cells and Bpa was encoded at multiple positions in the III-IV linker in the region of the quadruplet of amino acids “IFMT” which have been shown to participate in the fast-inactivation process.^93^ This approach allows for fast changes in the membrane voltage via whole-cell patch clamp techniques. Interestingly, while all four sites in the IFMT-locus tolerated Bpa encoding and produced macroscopic expression of rescued sodium currents, the phenylalanine position displayed conformationally dependent cross-linking phenotypes: when UV light was delivered to channels held a negative voltages, which bias resting “closed” channels, the resulting channels could still open but inactivation was removed with each bolus of UV light delivered. Conversely, UV shone on channels after they had inactivated appeared to lock them into an inactivated, nonconducting conformation. Taken together, the data support a model whereby movement around the IFMT locus is required for channels to enter and recover from the fast-inactivated conformation.

The utility of photoactivated cross-linkers is not limited to uses in electrophysiological studies. In studies of the K_ATP_ channel, cross-linkers were used to covalently immobilize subunits in various states, after which the cells were lysed and the channels analyzed via mass spectrometry.^94,95^ By comparing fragment sizes before and after photo-cross-linking, the investigators were able to show subunit interactions and compare these interactions between states. It should be noted that this method was used primarily to look at ligand-bound vs unbound states rather than voltage dependent states. These methods demonstrate the flexibility of the photoactivated cross-linker tools.

There are other cases in which UV-activated cross-linkers can be used for ion channel study. Bpa has been used as a photoactivator for the Orai1 component of the calcium-release-activated channel (CRAC).^96^ This work is derived from previous optogenetic work on this channel, that incorporated photoactivated signaling peptides into the necessary Orai1 cofactor STIM1.^97^ The noncanonical amino acid incorporation into Orai1 allows the channel to be opened without this cofactor, allowing more direct coupling of the UV pulse and channel activation. This technique can be utilized to precisely control the opening of this calcium channel in response to UV light.

## 4 Atomic Mutagenesis of Ion Channels

### 4.1 Charged Amino Acids

In ion channels and other membrane proteins, charged amino acids play important roles in structural contexts including key regulatory charge–charge interactions (salt bridges),^98^ ligand binding sites,^99^ and catalytic centers.^100^ For ion channels, the traditional method by which the functional role of these amino acids is determined is conventional site-directed mutagenesis combined with functional interrogation (electrophysiology). While convenient and informative, this approach is limited to the design features of the 20 canonical amino acids in terms of how specifically charge is altered, among other structural or chemical attributes.

The complications from a conventional mutation vary with the charged amino acid being studied. For example, the side chain of the basic amino acid arginine is quite large, and it features a distal guanidinium group not found in any other amino acid. Mutation of arginine to glutamine (by experimental design or clinical variant) not only neutralizes charge but also radically changes the side chain shape and introduces a potent H-bonding group, thus complicating the mechanistic interpretation of observed functional alterations. Similarly, the overall shape of lysine is not closely mimicked by any other natural amino acid, and thus, charge neutralization must be accomplished at the cost of side chain elimination (alanine) or profound changes in shape (glutamine). By contrast, the charge neutralization of acidic residues glutamic acid and aspartic acid by mutation to glutamine and asparagine, respectively, generally maintains amino acid shape. However, these mutations also add a potential hydrogen bond donor from the carboxyamide, which, depending on context, may complicate interpretation.^101^

To address these issues and attain enhanced insights into the functional roles of charged residues, it is possible to use GCE to substitute charged amino acids with near-isosteric unnatural analogs. Note that with a single exception discussed below, the identification of aminoacyl-tRNA synthetases specific for these analogs is not well developed. That said, multiple groups have successfully used the chemical tRNA misacylation/nonsense suppression method of GCE^29^ to encode the analogs in ion channels. We note that in our experience, the apparent incorporation rate of some polar amino acids via nonsense suppression (from the expression of engineered channels) is low^102^ compared to other types.^103^ The nature of this difference is poorly understood but may relate to side chain-dependent differences in hydrolysis and related stability of acylated tRNA.^42^ A similar principle may also explain why we and others have failed to produce stable suppressor tRNA acylated with specific polar AAs using a traditional chemical dinucleotide acylation scheme (e.g., natural arginine^104^).

#### 4.1.1 Basic Amino Acids

**Figure 5:**
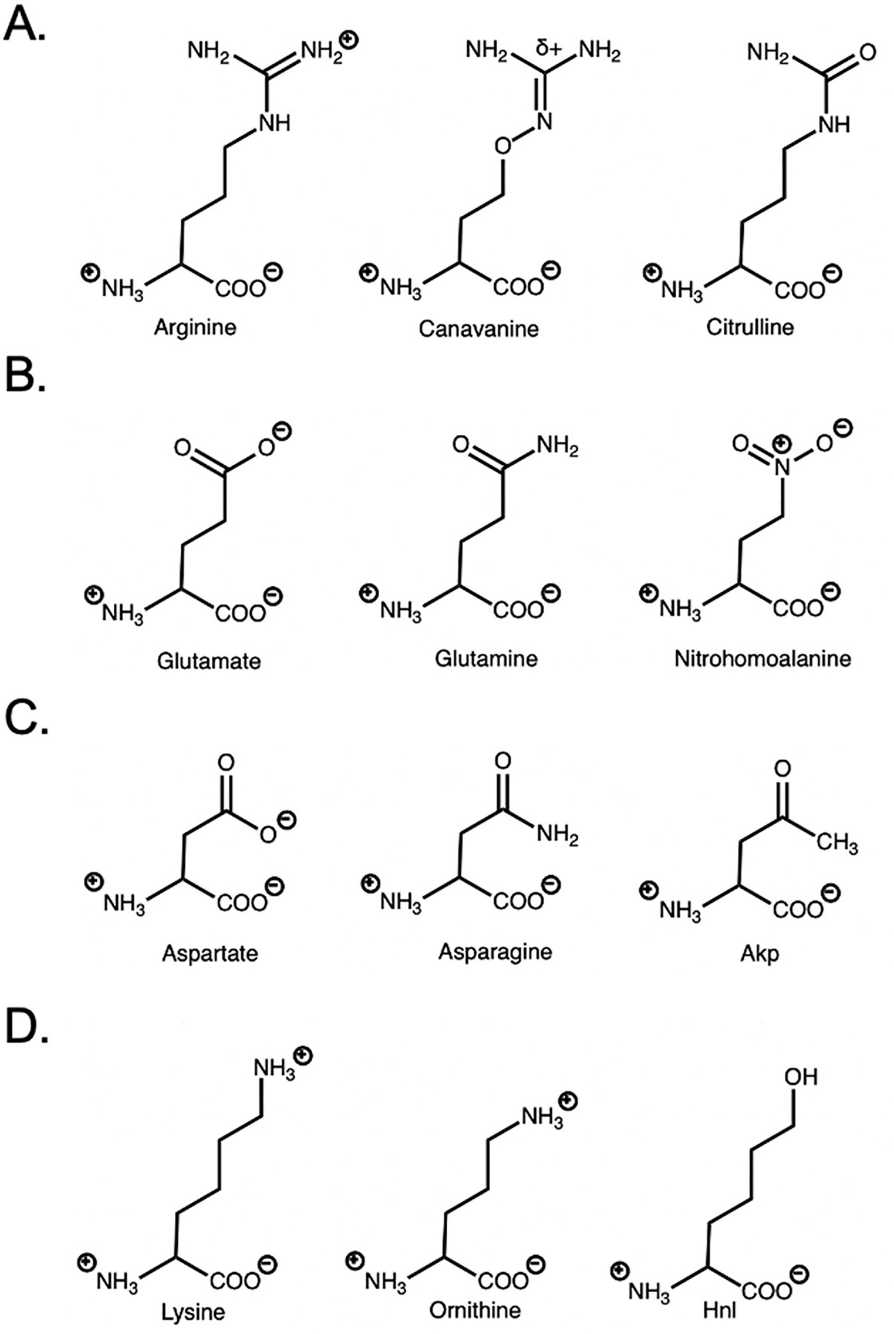
Unnatural amino acids designed as analogs of charged amino acids. All amino acids are expressed as assumed charges at pH 7.0. A., B., C., and D., show chemical structures of arginine, glutamate, aspartate, and lysine and analogs, respectively. Akp is an abbreviation for amino-4-ketopentanoic acid, and Hnl is an abbreviation for hydroxy norleucine.

The above challenges notwithstanding, multiple interesting analogs of arginine and lysine have been recently used to better understand the function and regulation of ion channels. We focused on the role of charge in the highly conserved arginine residues located at every third position on the S4 voltage sensor of voltage-gated ion channels.^104^ By isolating charge via citrulline substitution (Figure 5A), we found that positive charge at the fourth gating charge (R4) serves as a major stabilizing factor in the deactivated (closed) channel state,^104^ and that charge plays an outsized role, relative to that suggested by conventional mutation.^105^ Given the previously discussed limitations of the chemical misacylation system, the recent identification of a synthetase/tRNA pair specific for *photocaged* citrulline^106^ is an interesting and welcome advancement. Depending on application and context, use of the synthetase to encode caged citrulline within a protein, and then treatment with blue light for post-translation conversion to citrulline, may be preferred to the nonscalable injection of single *Xenopus* oocyte cells with chemical misacylated suppressor tRNA. As distinct from stable charge neutralization via citrulline, Lynagh and colleagues demonstrated the utility of substituting arginine with canavanine (Figure 5A), another near isostere of arginine whose charge is titratable (+1 → 0) within the physiological pH range. They encoded canavanine at a key arginine position (R76) within the ligand binding site of a model glutamate receptor ion channel.^107^ Increasing the pH of the extracellular recording solution by ∼ 2 units (expected to deprotonate and thus neutralize the side chain) caused a modest (2-fold) reduction in ligand affinity. These data suggested a lesser role for charge than was previously suggested by conventional mutation. We note that the interpretation of data from this approach is crucially dependent on the underlying assumption that the measured pH of the bath solution faithfully reflects the pH “sensed” by canavanine in its local structural environment. In structural contexts such as in Lynagh et al.^107^ (an aqueous extracellular ligand binding site), this is a relatively safe assumption. In contrast, for a buried and/or mobile arginine residue, one must consider the potential for the side chain p*K* to be influenced by surrounding amino acids and molecular compartments.

The Pless group recently circumvented the technical challenges intrinsic to encoding basic amino acids (and related analogs) via nonsense suppression using a method called “trans-splicing”: injection and *in-cell* intein ligation of a short synthetic peptide (see Section 2) within the ion channel expressed in *Xenopus* oocytes.^56,57^ They used this method to encode multiple lysine analogs at a critical position for gating of the ASIC ion channel.^58^ In this study, they found that positive charge and side chain length were both important to the role of this lysine in activation and desensitization.^58^ In the future, this general method (tandem protein trans-splicing of synthetic peptides bearing noncanonical amino acids) may enable the site-specific installation of many more unusual synthetic amino acids into membrane proteins.

#### 4.1.2 Acidic Amino Acids

Specific neutral analogs have been used to dissect the specific role of charge in glutamic acid and aspartic acid residues. For glutamic acid, 4-nitro-2-aminobutyric acid (a.k.a. nitrohomoalanine or Nha) represents a nearly isosteric, neutral analog without the complications of the foreign hydrogen donor group of the distal amide of glutamine (Figure 5B). This amino acid has been encoded at in place of glutamic acid at proposed “counter-charge” positions in the voltage sensors of K_V_^101^ and Na_V_^102^ to clarify the role of charge in gating. Likewise, illuminating examples of the atomic mutagenesis of aspartic acid with 2-amino-4-ketopentanoic acid (Akp) can be found in multiple studies of ligand-gated ion channels. Foundational work regarding the synthesis and encoding of Akp was accomplished by the Dougherty group;^108^ in this report, substitution of a critical aspartic acid in nAChR with Akp allowed relatively normal channel function, while asparagine substitution resulted in a large detrimental effect,^108^ suggesting that the native aspartate participates in an important allosteric hydrogen bonding network (which is disrupted by asparagine substitution). This example is informative not only in the structural understanding of this receptor but also potentially for the mechanisms of dysfunction for the myriad D → N human missense mutations responsible for channelopathies.

In a study by Pless et al., Akp and Nha were used to directly identify and test the functional role of two potential salt bridges (D148-R218 and E53-R218) in the glycine receptor (GlyR1) gating interface.^109^ Data from conventional mutation of both acidic residues suggests that either pair could play significant charge-based roles in gating.^110^ However, while neutralization of D148 with Akp caused noticeable effects on ligand binding, neutralization of E53 with Nha did not, strongly suggesting that a charge-dependent interaction between R218 and D148 (but not E53) is functionally dominant.^109^ More recently, in ASIC channels, a pore-lining glutamate was substituted with Nha; this isosteric neutralization caused a dramatic loss in the selectivity for Na^+^ over K^+^.^111^

### 4.2 Aromatic Amino Acids

In ion channel proteins, the aromatic amino acids phenylalanine, tyrosine and tryptophan are used to engage ligands, small molecules, and other aromatics and charged side-chains.^99,112−114^ Cation−π interactions occur between a positively charged cation and an aromatic π system. The cation can come in a variety of forms: neurotransmitters, small molecules (i.e., drugs), toxins; all systems that form “fuzzy” cations with relatively weak hydration energies. There is also some growing evidence that suggests that such interactions may play roles with membrane lipid bilayer headgroups, or even from other proteins, in the form of the cationic amine or guanidium moieties of lysine or arginine. The quantitative evaluation of a putative cation−π interaction requires the use of noncanonical amino acids because there are no easily substituted side-chains that allow the experimental determination of cation−π binding energy. For instance, replacement of a given aromatic by alanine or leucine may provide low-resolution information about the side-chain bulk or hydrophobicity of the aromatic, but in terms of cation−π binders, these substitutions are essentially “all or none” in their experimental utility. The loss of a particular ion channel function or binding event upon replacement of an aromatic group with a nonaromatic amino acid *could* indicate the presence of a cation−π system, but such a result could also equally be due to the loss of steric or hydrophobic forces. In order to circumvent this experimental limitation, the Dougherty and Lester laboratories formalized an experimental system whereby serially fluorinated versions of phenylamine or tryptophan are encoded into an expressed ion channel in the *Xenopus laevis* oocyte expression system.^114^ The study of cation−π interactions, primarily in ion channel proteins, has been grounded with this so-called fluorination strategy, whereby the hydrogen atoms of the aromatic of interest are serially replaced with fluorine. The experimental strength of this approach derives from the fact that fluorination of the π-donor aromatic ring reduces the overall arene quadrupole moment in a linear manner.^114^ At the same time, the fluorine atom is roughly isosteric to hydrogen in this context, thus leaving the overall size of the aromatic modestly changed. Thus, serial fluorination of an aromatic can be used as a framework for the hypothesis, with binding energy then approximated from an effect on channel function. For example, cation−π systems featuring chemical compounds like neurotransmitters or ion channel blockers have been inferred and quantified by directly measuring the apparent loss of ligand efficacy in an electrophysiological determination ion channel conductance. This benchtop laboratory method is further enhanced through a combination of computational methods that can be easily married with structural data that is now available for most classes of ion channel proteins. This systematic approach represents a gold-standard for subatomic resolution pharmacology for cation−π systems and has been successfully applied to more than 30 receptor and ligand types.^114^ Aromatic–aromatic and aromatic–Lys/Arg interactions have been far less characterized with noncanonical amino acids in ion channels, although recent advances in available modes of encoding will likely open new advances on this front.^115^

#### 4.2.1 Using Noncanonical Amino Acids Enables Subatomic Pharmacology in Postsynaptic Receptors

Cation−π interactions rely on the negative quadrupole moment generated by the electron densities of an aromatic group. Thus, to examine the basis for such an interaction, one must carefully manipulate the subatomic location of electrons that would otherwise form the negatively charged electrostatic center of the aromatic side-chain. Serial fluorination allows such an approach and has been applied to the binding chemistry of agonists and pharmacophores of neurotransmitters receptors. The first characterized natural cation−π system was found to be between the cationic amine of the neurotransmitter acetylcholine and the aromatic-box motif that is formed by aromatic side-chains expressed on the exofacial domain of the ligand-gated channel. This noncovalent yet incredibly precise scaffold supports fast synaptic communication between the presynapse, where acetylcholine-filled vesicles dock and then fuse, and the postsynaptic membrane where neurotransmitter receptors are known to cluster. In a landmark paper, the Dougherty and Lester laboratories encoded fluoro-aromatic derivatives in the AChR aromatic-box motif to demonstrate the existence of a cation−π interaction between the prototypical nicotinic acetylcholine receptor and the quaternary ammonium group of the agonist, acetylcholine.^116^ To further characterize this interaction, *ab initio* quantum mechanical predictions were made for a simplified cation−π binding system. Notably, these predictions were found to closely match the recorded EC50 values for acetylcholine at the receptor for a series of fluorinated tryptophan derivatives at position W149 in the receptor. Interestingly, this linear correlation upon fluorination was only found for W149 in the AChR α-subunit and not other nearby aromatic box residues, suggesting that only one of the aromatics forms a bona fide cation−π with acetylcholine. Notably, the binding pose between W149 and ACh that was initially discovered through chemistry, electrophysiology, and *ab initio* computation has since been structurally validated with X-ray crystallography^117^ as well as cryo-electron microscopy.^118^

Given the conservation across multiple receptor classes and protein sequences, this site has been termed TrpB in the literature based on its position in the aromatic box motif. Acetylcholine receptors are also expressed at the neuromuscular junction in skeletal muscle, where they couple peripheral nerve impulses to coordinate muscle contraction. Nicotine is a well-known AChR agonist that is also known to bind in the brain AChR binding pocket as a primary event in nicotine addiction. However, if nicotine also had a high-affinity interaction with skeletal muscle AChRs then the use of tobacco products would result in potent and potentially fatal muscle contraction (via uncontrolled activation of skeletal muscle AChRs). Such a mechanism would be not dissimilar to nerve agents that inhibit acetylcholinesterases^119^ (both resulting in excessive activity of AChRs in the neuromuscular junction). Given that TrpB is conserved between muscle and brain ACh receptors, nicotine could feasibly participate in such a high affinity interaction. To solve this molecular puzzle, the Dougherty and Lester laboratories first studied cation−π binding in the brain α4 β2 brain receptors thought to underlie nicotine addiction and confirmed a strong cation−π interaction between nicotine and TrpB in these receptors. Surprisingly, they then showed that TrpB in *muscle* AChRs does not form a cation−π interaction with ACh.^120^ How is this possible? It was suggested by the authors that a key amino acid variation between α4β2 and muscle-type isoforms located nearby may influence the shape of the aromatic box, thus allowing nicotine to interact more strongly with TrpB in the brain receptor but weakly in the muscle AChR. This result serves as a real life example of the importance of cation−π geometry between the π-system and the cation. It is also difficult not to note the evolutionary significance of such a minor difference between skeletal and brain receptors, one that would turn a compound like nicotine from a potential neurotoxin to an addictive neuro-stimulant.

Further, while TrpB is the sole aromatic of five that forms strong cation−π interactions with ACh and nicotine, it has also been shown that a second aromatic, TyrC2, forms cation−π interactions with the AChR agonists metanicotine, TC299423, varenicline, and nornicotine. A common structural feature of these agonists, and a distinction from ACh and nicotine, is a protonated secondary amine that provides the cation for the cation−π interaction. These results indicate a distinction in binding modes between agonists with subtly different structures, which may provide guidance for the development of subtype-selective agonists of nAChRs. For example, the smoking cessation drug cytisine engages the Α2β4 nAChR subtype via an intricate network with multiple cation π interactions.^121^ The nicotinic pharmacophore binding site has also been shown to display a variety of chemical features, including hydrogen bonding with main-chain amides.^122,123^

It has been further proposed, using similar methodology, that other postsynaptic receptors use cation−π interactions with neurotransmitters.^124^ These additional members of the Cys-loop receptor family include serotonin, glycine, and GABA receptors, all of which use aromatic residues to engage ligands; although in place of the TrpB residue, the glycine and GABA-A receptor binding sites use phenylalanine and tyrosine, respectively. Cation−π interactions have been proposed between these aromatics and positively charged moieties in relevant ligands.^125−128^

It is useful to interpret these many functional studies in light of recent atomic and near-atomic resolution structures of ligand-bound cys loop receptors. As discussed above and illustrated elsewhere, for the nAChRs, structures have revealed ligand poses in the “aromatic box” that are generally consistent with an *en face* interaction between the functionally implicated, conserved tryptophan in binding site loop B (so-called “TrpB”) and the cationic moiety of acetylcholine and analogs.^99,117,118^ By comparison, for some of the related pentameric channels, the available structures suggest that aromatics engage cognate ligands by using diverse chemistries tailored to the energetic and steric requirements of functional groups.

**Figure 6:**
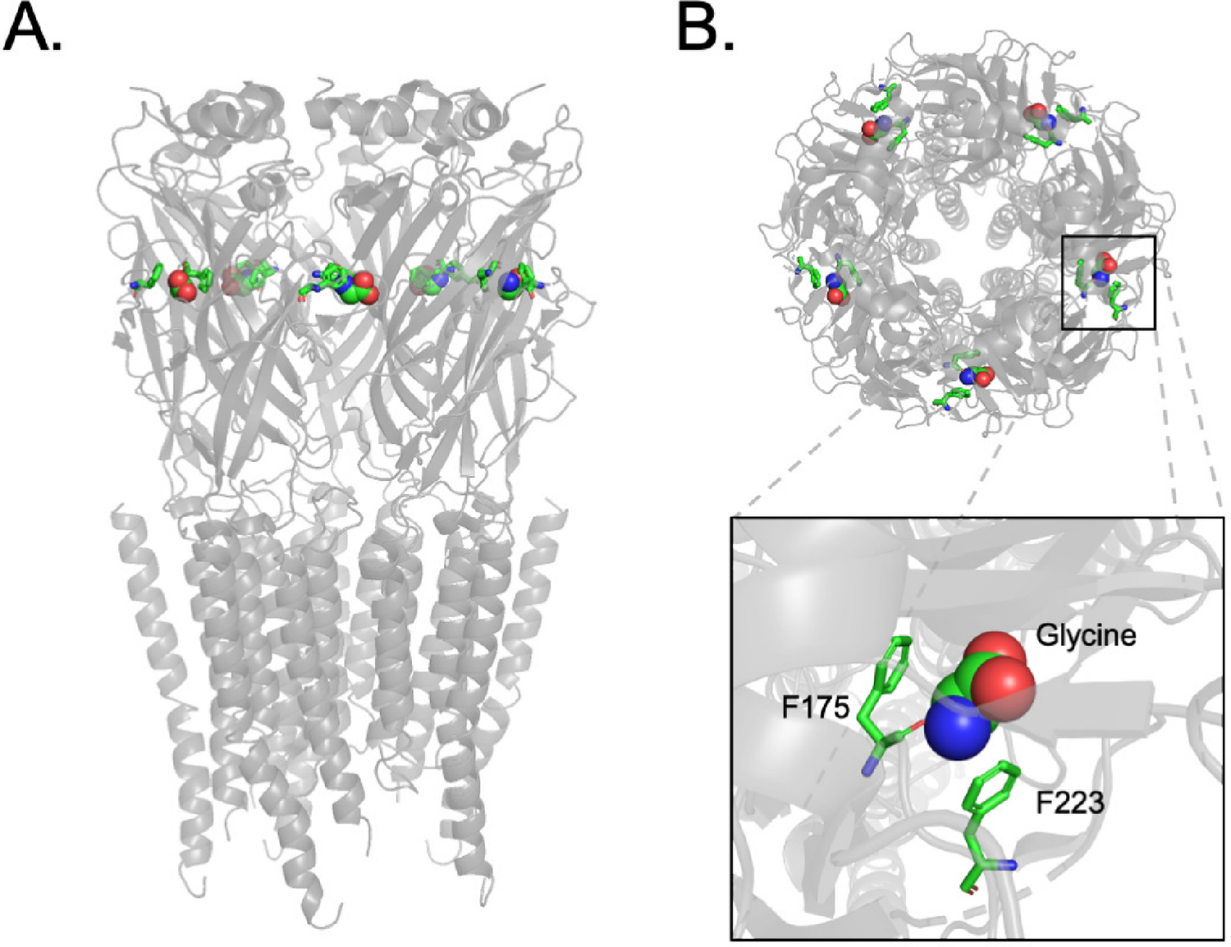
Phenylalanine residues in a cation−π interaction with the ligand in the glycine receptor (GlyR). A. Side view of homomeric GlyR (*danio rerio*) (pdb 6PM6). Cryo EM structure from the channel extracted in native lipid nanodiscs via SMA. Key phenylalanine residues and glycine ligand are shown in color. B. Top view of the same structure, with inset showing close-up of the positioning of the loop B Phe (F175) and loop C Phe (F223) relative to the amide group of the ligand.

In the homomeric GlyR, the ligand binding pocket is replete with aromatics, including multiple phenylalanine residues.^129^ Functionally, “PheB” (homologous to TrpB in nAchR) displayed the most straightforward fluorination effect (a linear decrease in affinity with increased fluorination), leading to the interpretation that this cation−π interaction dominates electrostatics.^128^ That said, in the Cryo-EM structures, a loop C Phe residue (“PheC”) is adventitiously positioned for an *en face* cation pi interaction with the glycine amide. PheB is also nearby, posed to interact with the ligand cationic group at least in part via its backbone carbonyl^130,131^ (Figure 6). Looking back at the functional (fluorination) data for GlyR, we note that large effects on ligand potency were observed for unnatural substitutions at PheC, although the effect of increased fluorination was not nicely linear.^128^ The high-resolution structural data now in hand may help explain these functional results via specific compensatory or exacerbating interactions that exist nearby. Of note, the structural biology of the GABA-A binding site positions beta-subunit tyrosines on loops B and C in a very similar fashion to that of PheB and PheC in GlyR.^132^

The ionotropic serotonin receptor 5-HT_-3A_ possesses an aromatic ligand binding pocket highly homologous to nAChR, including a Trp on loop B.^124^ Interestingly, in bound structures, serotonin and related ligands are positioned with their cationic moieties pointed away from this TrpB,^133^ whose fluorination linearly reduces ligand potency in this channel.^125^ A complete understanding of these functional and structural data may require invoking alternative physical mechanisms for the effect of TrpB fluorination; for example, fluorination may directly disrupt the electrostatics of a favorable T-shaped interaction with serotonin. Overall, it is evident that the power of the aromatic fluorination method to functionally test aromatic interactions with ligands is enhanced, and overall mechanistic understanding is enriched, when informed by relevant structural biology data.

#### 4.2.2 Cation−π Interactions in Voltage-Gated Ion Channels: Ligands, Therapeutics, Toxins, and in Structural Contexts

Voltage-gated cation channels shape electrical signals in the excitable cells of nerve and muscle.^5^ They control the permeation of their nameake ion by sensing the transmembrane voltage and quickly communicating this information to an intracellular component of the protein, which serves as a “gate” by which ion flux is controlled. Voltage-gated channels are large multispan transmembrane proteins that share a similar architecture: four homologous membrane embedded domains which surround a central aqueous permeation pathway and ionic selectivity filter. The four peripheral domains comprise the first four transmembrane segments (S1–S4) and house the core module of the voltage-sensing mechanism. These channels are essential for a variety of functions that are synonymous with human experience: memory and learning, proprioception, sensing of temperature and pain, mobility, and cardiac function. For this reason, they represent high-value targets for the development of therapeutics. Common examples include some ion channel medicines, which act as physical channel blockers that occupy the central aqueous pathway and thus prevent the flow of ions. One possible mechanism for this blockade is that cationic functional groups on therapeutics mimic a partially or fully hydrated version of the permeant sodium or potassium ion, thus gaining access deep within the ion pathway. But due to steric restrictions, the therapeutic cannot fully cross the membrane and becomes stuck. Sodium channel blockers, such as local anesthetics, bind to an aromatic laden site within a water-filled inner vestibule. The resulting loss of inward sodium conductance results in a reduction in cellular excitability, an outcome that has been used for the treatment of pain, cardiac arrhythmia, and epilepsy. Similarly, given the biological importance of voltage-gated channels, evolution over eons has honed small molecules and peptide toxins which engage these channels as molecular targets in predator/prey relationships. Indeed, many venomous creatures possess ion channel modulators in their venoms. For instance, the small molecule tetrodotoxin (TTX) can suppress sodium channel activity in skeletal and nervous system channels to effectively paralyze prey for future consumption (e.g., blue ringed octopus),^134^ or temporarily paralyze an aggressor for defense (e.g., pufferfish^134^). In other cases, complex peptide mixtures found in the venoms of in jellyfish, spiders, scorpions, and snakes can open peripherally expressed sodium channels that reside in pain pathways, thus promoting the *perception* of extreme pain—a clear warning that will likely be remembered.^134^

TTX is a cationic molecule that derives its potency from a guanidinium moiety that is needed for sodium channel blockade. First discovered in the gonads of pufferfish (*fugu*),^135^ TTX has been studied extensively for its ability to potently inhibit sodium channels.^136^ TTX is produced by symbiotic bacteria and the host develops resistance via evolutionary modification of endogenous (host) sodium channels^137^ or through toxin sequestering.^138^ Of the nine human voltage-gated sodium channel isoforms, two, Na_V_1.5 in the heart and Na_V_1.8 in peripheral nerves, are essentially insensitive, a feature that has long been used to characterize native sodium currents.^139^ TTX inhibition is also “use-dependent,” that is, channels that are open more frequently have an apparent higher affinity for TTX.^140^ A common feature of all TTX sensitive channels is the presence of an aromatic tyrosine that sits at the external entrance of the selectivity filter. Removal or replacement with a nonaromatic site chain will render these channels TTX insensitive. By encoding fluoro-Phe noncanonical amino acids at this site it was revealed that TTX relies heavily on a cation−π interaction for high-affinity blockade.^141^ Interestingly, this study for the cation−π-binding component of block was similar for resting or active channels. Given the intrinsically strict orientation requirements of the cation−π interaction, the fact that it remained unchanged suggests that the TTX molecule stays fixed at the entrance to the channel in both resting and open channels. One likely explanation for the increased affinity in open channels yet limited movement in the TTX binding pocket is the loss of positively charged sodium ions in the nearby (∼3 Å) selectivity filter. It has been proposed that as these ions unbind from open channels, there is a loss of cation–cation repulsion between Na^+^ and TTX; although other mechanistic possibilities exist.^142^ The available high-resolution structures of TTX-bound cockroach Na_V_ (PDB 6A95)^143^ and TTX-bound human Na_V_1.7 (PDB 6J8J)^144^ indicate that the guanidinium moiety of the TTX could be within distance to form a cation−π interaction with the pore-lining aromatic. However, it is worth considering that such structures are obtained in the absence of a transmembrane electric field, and thus, the possibility exists that these channels may visit, or represent, protein conformations of unknown significance. In the case of TTX binding to the extracellular vestibule, for example, it is known that depolarization-driven, voltage-dependent transitions in the voltage-sensors, primarily DIV, can promote rearrangements in the selectivity filter.^145^

Sodium channels are also the targets of local anesthetic molecules, which include antiepileptics and Vaughan-Williams Class Ia-c antiarrhythmic agents. These drugs share a common architecture with a primary, secondary, or tertiary amine and short tether and then often an aromatic group with a variable number of substituents. Unlike TTX, local anesthetics are intrinsically nonselective in their inhibition of the nine human sodium channel isoforms. However, their clinical utility relies on their ability to target overactive channels that typify the electrical hyperexcitability of arrhythmic and epileptic tissues. Through this mechanism, open and inactivated channels have a 10–50-fold higher affinity for a given local anesthetic agent; thus, the drug preferably inhibits channels in pathologically excitable tissues and spares those channels in less excitable tissues. The drug binding site is found in the inner vestibule of the channel, a water-filled cavity just below the selectivity filter that is lined by two aromatic residues along a pore-lining α-helix. Using noncanonical Fluoro-Phe derivatives, it was first shown that the use-dependent inhibition of voltage-gated Na_V_1.4 channels by the local anesthetic lidocaine relies on a cation−π interaction with the upper aromatic, Phe1579, but not the lower site.^146^ Interestingly, this study found that the low-affinity “tonic” block was unaffected by aromatic fluorination.^146^ In a second study, the cation−π binding abilities of representative Vaughan-Williams Class Ia-c antiarrhythmic agents were examined in cardiac Na_V_1.5 channels, the clinical target of this class of therapeutics.^147^ Here, the data show, somewhat unexpectedly, that the class 1b drugs lidocaine and mexiletine rely on cation- π energetics for use-dependent inhibition, but their chemical congeners in the 1a and 1c class categories do not. And like Na_V_1.4, none of the drugs showed a cation−π preference for the low affinity block of resting channels. Taken together, the data suggest despite the similar chemical nature of local anesthetics, including a shared cationic amine, these compounds rely on a variety of binding modes to effect channel function. Further, the state-dependent nature of the class 1b cation−π interaction suggests the possibility that the pore-lining helix undergoes a translation or rotational movement that orients the aromatic side-chain toward the inner vestibule in open and inactivated channels, and away in closed channels. This possibility will require examination with more sophisticated approaches to inform on such fine-grained motions.

Voltage-gated potassium channels (K_V_) establish the negative resting membrane potential in cells by selectively enabling K^+^ ions to leave the cell down their electrochemical gradient. These channels share a highly potassium-selective central pore and are gated (opened) by positive (*de*polarizing) changes in the membrane potential. Like TTX with Na_v_, the quaternary ammonium tetraethylammonium (TEA), blocks K_V_ channels when applied extracellularly^148^ and does so in an isoform dependent manner.^149^ Inspection of the protein sequences just extracellular to the selectivity filter between these channels reveals that TEA sensitive channels present a conserved aromatic side-chain.^150^ Because K_V_ channels are homotetramers, this arrangement results in an outward facing four-sided aromatic cage formed by the side-chains of aromatic side-chains. Site-directed mutation of this site to nonaromatic side-chains disrupts the high-affinity blockade by TEA. With this motivation in hand, it was then shown using F-Phe encoding at this site, that TEA extracellular blockade relies on a cation−π interaction^151^ and this may promote a slow form of channel inactivation.^152^ Interestingly, the available potassium channel structures from the thermophile, *KcsA*, depict this aromatic group in two orientations. The WT KcsA channel shows an exofacial TEA site buttressed by four tyrosine side-chains (Tyr82 in KcsA, PDB 2BOC).^153^ However, upon close inspection, the side-chain orientation is positioned as an en-edge, with the negative quadrupole on the face of the aromatic oriented away from the TEA cation. In contrast, the structure of a mutant form of KcsA (Glu71Ala) alters channel inactivation gating and finds Tyr82 reoriented, now in an *en-face* position with the four aromatics facing directed toward the expected position of bound TEA (PDB 2ATK).^154^ Thus, these structures suggest that a conformational mobility exists in the external TEA site, and this movement would be predicted to impact TEA affinity as well as binding chemistry. This aromatic is poorly conserved in the Kv channel-types used structural studies, thus barring a direct comparison, as is the case for the Kv1.2–2.1 paddle chimera,^155^ Shaker Kv channels,^156^ and Kv2.1.^157^ However, similar to prokaryotic channels, this position shows significant conformational mobility, suggesting that local movements would impact the orientation of channel types with an aromatic present.

K_V_ channels are also clinical targets because compounds that open these channels have the potential to quelle electrical hyperexcitability by promoting more negative resting membrane potentials and less action potential firing. Retigabine, for instance, is a potent antiepileptic agent that selectively activates neuronal K_V_7 channels by inducing a hyperpolarizing shift in voltage gating. Using noncanonical amino acid mutagenesis, Kim and colleagues investigated the role of a tryptophan side-chain that is only present in drug-sensitive isoforms and where mutation of this side-chain (even to other aromatics) results in a drastic loss of drug affinity.^158^ K_V_7 channels bearing encoded F-Trp substitutions at this site displayed normal function, but each added fluorine atom increased retigabine potency, a result that is at odds with a simple cation−π binding arrangement. To better understand this mechanism the authors synthesized an encoded a new Trp analogue, termed IND, whereby the indole nitrogen group had been subtly moved such that the amide H-bond was removed while sparing side-chain aromaticity. Surprisingly, IND encoded channels were completely resistant to retigabine opening as well as to a variety of similar K_V_7 agonists. Thus, in this example, noncanonical amino acid mutagenesis was uniquely able to reveal a novel form of ion channel pharmacology: H-bonding from an essential Trp indole group was indispensable for drug affinity and efficacy. This channel class and drug binding site are currently the focus of intense pharmaceutical development for epilepsy, pain, and cardiac arrythmia.

#### 4.2.3 Investigating Cation−π Interactions in Structural Contexts within Proteins

While experimental evidence abounds for cation−π interactions between ligands and aromatic receptors in ion channels, cation−π interactions between the side-chains in channel proteins remain largely unexplored. Multiple computational studies have found that such interactions should be common in the proteome. In one example, energetically significant cation−π interactions were computationally assessed from a data set of ∼600 soluble protein structures.^159^ This analysis indicated that of the ∼230,000 residues that were considered (inclusive of all amino acids), almost 14,000 interacting pairs of Arg/Lys side-chains were found with appropriate proximity and geometry to aromatic Trp/Phe/Tyr residues to form cation−π interactions. Notably, as many as one-fourth of these pairs were predicted to form significant cation−π interactions, > −4.0 kcal/mol. More recently, a computational study of the rapidly growing database of membrane protein structures solved by Cryo-EM found a similar result: cation−π interactions should be common in membrane proteins, including ion channels.^99^

The characterization of some of these putative protein–protein cation−π interactions with noncanonical amino acid mutagenesis has been challenging.^160^ One notable example is the study of the gating charge transfer center found in the voltage-sensing domains (VSDs) of K_V_, Na_V_, and Ca_V_ channels.^161^ VSDs sense and respond to transmembrane voltage by shuttling Arg and Lys side-chains along the α-helical S4 segment from inside to outside the cell to control the conductance status of the pore domain. VSDs also focus the trans-bilayer electric field across a narrow gap in the VSD that separates the extracellular and intracellular sides of the bilayer. Whereas the lipid bilayer is ∼40A thick, this focused gap tightens to ∼5–7A, with a highly conserved Phe residue at the core of this gap. Thus, as the VSDs respond to voltage, Arg and Lys side-chains are rapidly transported past the side-chain of the so-called “Phe gap”, where the aromatics could act as catalysts for cation transfer across an otherwise hydrophobic membrane core. To study this phenomenon with noncanonical amino acids, the Dougherty/MacKinnon and Ahern laboratories separately encoded F-Phe derivatives at the VSD Phe-gap position in *Shaker* potassium channels that were subsequently assessed for function by electrophysiology in *Xenopus* oocytes.^161,162^ Both groups found that serially increasing fluorine atoms did not result in a linear shift in kinetics (open/close rates) or equilibrium gating parameters, suggesting that no such catalytic cation−π interaction exists at this site. However, encoding a Trp at the Phe-gap was shown to induce a cation−π interaction, likely with the cationic side-chain on S4 (Lys374).^162^ One possible explanation for this result is that any such cation−π interaction is required for protein folding and trafficking but not function.

In the context of lipid bilayers, putative cation−π interactions have been identified as lipid-specific anchors for phosphatidylinositol-specific phospholipase C (PI-PLC);^163^ whereby PI-PLC interacts with phosphatidylcholine (PC) headgroups through cation−π interactions with tyrosine residues. A similar interaction has been identified in the pentameric ligand gated ion channel family.^164^ In the human large-conductance potassium channel BK, similar interactions have been found between a cytoplasmic tyrosine and lipid headgroups,^165^ possibly via charged choline groups. Future and ongoing studies will begin to reveal the energetic contributions of these interactions to ion channel function, modulation, and pharmacology.

In addition to supporting cation binding, aromatic–aromatic encounters can occur between neighboring side-chains or with aromatic moieties of ligands. In one ion channel example, noncanonical amino acids have been used to demonstrate aromatic interactions between a ligand and aromatic side-chains in H_V_1 proton channels.^166^ The significance of the interactions largely relies on how the two aromatic moieties engage one another through either a planar/parallel stacking or perpendicular/T-shaped pose. However, to date, experimental examination of such interactions has been limited mostly to small model proteins.^167^ This outcome may be due, in part, to the limited number of proteins that can be studied in the *Xenopus laevis* oocyte expression system. For these proteins, the development of new tools for encoding of fluorinated aromatics will likely open new paths toward experimentation. One important step in this direction was recently made with the identification of an evolved synthetase/tRNA pair from the pyrrolysine class that allows for selective encoding of fluoro-phenylalanine noncanonical amino acids in prokaryotic and mammalian expression systems.^115^

### 4.3 Backbone Substitutions to Dissect Main-Chain Hydrogen Bonds

Compared to altering amino acid side chain via simple point mutation of relevant codons, manipulating the backbone groups of a polypeptide is complicated by the strict biochemical requirements for *in vivo* elongation.^168^ Proline mutation may perhaps be considered a facile, course-grained approach to backbone mutation, but this substitution simultaneously changes peptide secondary structure and side chain, in addition to altering the chemistry of the α amide.^169^ Accordingly, the structure–function study of the polypeptide main-chain is particularly well paired with chemical biology (GCE) methods enabling the encoding of noncanonical amino acids with specific backbone alterations.^170^

Myriad backbone chemical substitutions are technically achievable through solid phase peptide synthesis (SPPS),^171^ while a small but increasing number have been shown to be compatible with the PURE *in vitro* translation system, which is based on prokaryotic ribosomal translation.^172^ To our knowledge, thus far only one type of modification—α amide-to-ester substitution—has been found to be compatible with *in-cell* ribosomal translation (and thus “encodable” through nonsense suppression). The encoding of these α-hydroxy acids, usually in place of their cognate amino acids, has been used extensively in the study of ion channel structure, function, and pharmacology via injection of chemically misacylated amber suppressor tRNA^29^ for nonsense suppression.

Amide-to-ester mutations have been incorporated in several ion channels to disrupt intrahelical backbone hydrogen bonding. The method was first leveraged to make mutations at multiple positions within the transmembrane helices of the nicotinic acetylcholine receptor (nAChR).^173^ Interestingly, disrupting these putative H-bonds had varied effects on channel gating (i.e., both loss *and* gain-of-function) via mechanisms that are still not well understood. More recently, we incorporated α-hydroxy acids along the voltage sensing helix (S4) of the model eukaryotic K_V_ channel *Shaker*.^103^ These amid-to-ester mutations generally perturbed channel gating, and the degree of effect was highly position-dependent. A functional “hot spot” was found at the transition between the putative α helical (*i*+4) and 3(10) (*i*+3) helical segments of the activated S4, where H-bond scission elicited large shifts in channel gating. A mechanism was suggested by molecular dynamics simulations showing that the structure of this transition was reliant on H-bond integrity to a far greater degree than the fully α-helical regions in the voltage sensor. This same type of chemical biology manipulation has been used successfully to directly test the roles of backbone amide groups in the pharmacology of ion channel ligands. For example, in the α4β2 nAChR channel, substitution of a backbone amide in the binding site to ablate a proposed H-bond interaction of the pharmacophore caused substantial (∼10-fold) reductions in the affinity of acetylcholine and nicotine.^174^

An additional use of backbone mutations in ion channels has been to study ion channel selectivity: the degree to which the channel pore discriminates among different ions.^175^ For example, the Voltage-gated Cation (e.g., Na_V_, Ca_V_, K_V_) channels display strong preference for their namesake ions,^176^ while the CLC voltage-gated chloride channels strongly prefer anions over cations.^177^ Backbone groups often feature prominently in the structural biology of the so-called selectivity “filters” of these channels, wherein they interact directly with permeating ions.

In CLC channels, Cl^–^ ions proceed through a selectivity filter comprised of three successive anionic binding sites^178^ and involving a host of backbone amide groups contributed by multiple amino acids.^179^ Recently in a study by Leisle et al., these amide groups were systematically mutated to esters in representative CLC channels.^180^ Overall, ester mutation suggested roles for backbone amides in selectivity that were dependent on the position within the external and central anionic binding sites and further modulated by the protein background (with or without a canonical “glutamate gate”). Of note, at several positions, amide-to-ester mutation elicited a higher-than-WT relative permeability of NO_3_^–^ compared to Cl^–^ (or Br^–^), suggesting significant degradation of the selectivity mechanism.

Potassium channels feature a conserved selectivity filter lined with backbone carbonyl groups spaced perfectly for energetically favorable permeation of a dehydrated K^+^ ion.^181^ Backbone carbonyls have also been proposed to play featured roles in the cation selectivity of ASIC via a structural motif common to its channel superfamily.^182^ In both types of channels, amide-to-ester mutation has been used to study mechanisms of selectivity,^111,183^ leveraging the principle that the amide-to-ester substitution should reduce the electronegativity of the neighboring backbone carbonyl group of interest.^184,120^ In both studies, the effects of amide-to-ester substitution on the selectivity were minor (in ASIC, actually an increase in sodium-to-potassium selectivity). This could be due to the possibility that native/near electronegativity of the given carbonyls is not necessary for ion selectivity; or possibly, that in these structural contexts ester substitutions are not having the intended effects on relevant dipoles.

The present literature highlights the need for new tools to manipulate backbone (particularly carbonyl) chemistry in large, cotranslationally folded proteins such as ion channels. This may be accomplished through major future technical advances in membrane protein trans-splicing^56^ or the incorporation of dipeptides as a single ribosomal event.^185^ That said, amide-to-ester substitution via α-hydroxy acid incorporation remains a powerful and seemingly still underutilized tool to directly test the chemical contributions of the NH backbone group in the myriad structural contexts where it has been proposed to play an important role.

## 5 Fluorescent Amino Acids in Ion Channels

Fluorescent techniques have been widely used to study ion channel localization^186^ and the conformational changes associated with channel function and regulation.^187^ For simple studies of localization, the most popular method is genetic fusion of a fluorescent protein, such as GFP.^188^ Because GFP is a somewhat large, (>20 kDa) β-barrel protein module, care must be taken in the location of the fusion (N-terminal, C-terminal, internal^189^). The addition of flexible peptide linkers between the channel and fluorescent protein is often also required to maintain normal function.^189^ Recently, significant effort has been undertaken to achieve high specificity labeling of proteins using a smaller molecular biology footprint than that of GFP. A family of such methods utilizes the introduction of short peptide sequences for labeling under mild chemical conditions. These methods include the FlaSH tag (arsenical hairpin),^190^ Alfa tag (nanobody),^191^ and the bungarotoxin-binding site tag,^192^ all of which generally require aqueous access to the labeling position.

**Figure 7:**
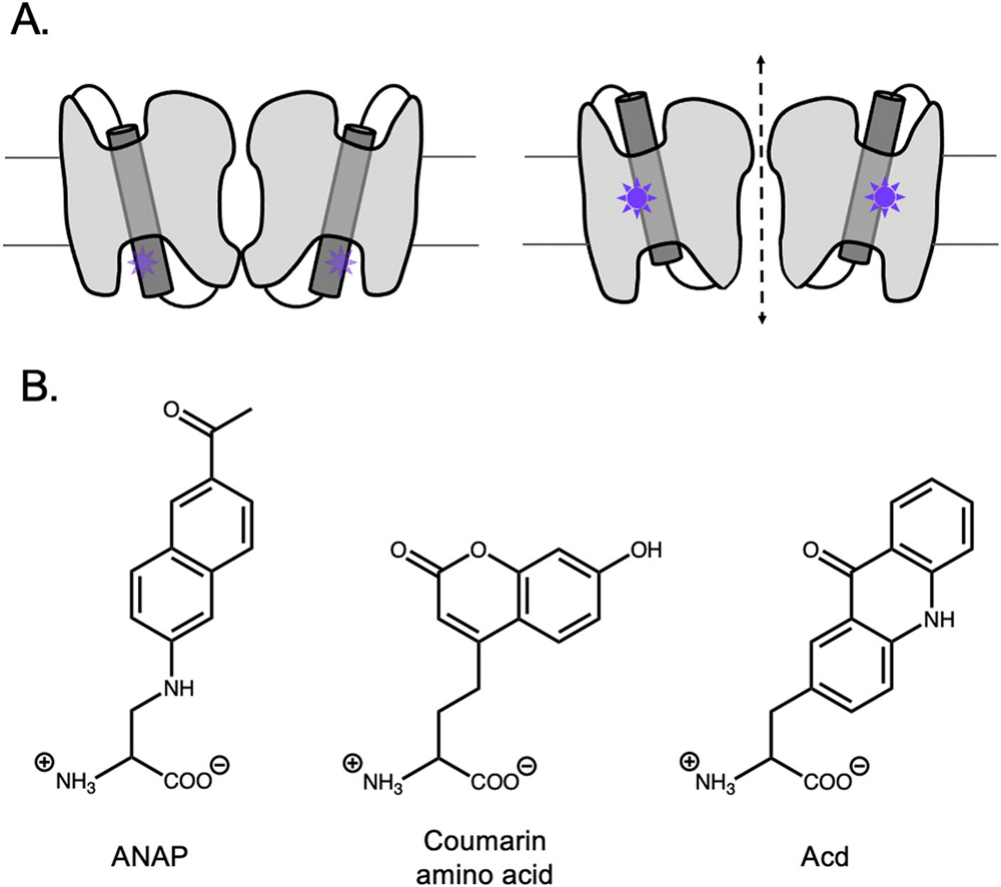
Use of fluorescent unnatural amino acids in studies of ion channel structure and function. A. Schematic example of voltage clamp fluorometry using an environmentally sensitive fluorophore attached to the voltage sensing helix of an ion channel. B. Chemical structures of three fluorescent unnatural amino acids encoded by specific orthogonal tRNA/synthetase pairs. Chemical names of the amino acids are for ANAP: 3-(6-acetylnaphthalen-2-ylamino)-2-aminopropionic acid; for Coumarin: L-(7-hydroxycoumarin-4-yl)ethylglycine; and for Acd: acridon-2-ylalanine.

To effectually report on the (often subtle) conformational changes associated with ion channel gating, it is necessary to utilize strategies that allow extreme proximity between the chromophore and site of interest in the protein. To achieve this, a commonly used method involves labeling introduced cysteine residues with thiol-reactive, environmentally sensitive fluorophores.^193,194^ As with the labeling approaches discussed above, this method generally requires aqueous access to the site of interest.^195^ This approach has been applied elegantly as “voltage clamp fluorometry” (Figure 7A) in studies probing the kinetics of movement of key ion channel domains; especially in how these movements relate to the elaboration of specific functional states.^196−200^ However, the method lacks versatility (movements are observed only if polarity around the probe changes) and specificity (background cysteines on the protein of interest or other proteins could be labeled). Moreover, they rarely provide any meaningful spatial coordination. To measure relative distances between points on ion channels, several different configurations of fluorescence resonance energy transfer (FRET)^201^ have been used. FRET utilizes the steep, Å-scale distance dependence for probes to donate and accept Förster resonance energy.^202^ Ideally, the fluorescent donor and fluorescent (or nonfluorescent^203^) acceptor probes will be chosen for favorable FRET radii^203^ in the structural context of interest. However, the magnified insights of FRET come at the cost of the formidable technical challenges associated with achieving dual-specific labeling at the intended structural positions and with the intended fluorophores.^204^ Consequently, relatively few ion channel studies have utilized FRET, and these studies have generally required methodological compromises that substantially affect interpretation.

Significant effort has been dedicated to leveraging GCE in the development of fluorescence techniques. For example, there are multiple reports describing the encoding of a range of environmentally sensitive and insensitive fluorophores as noncanonical amino acid analogs via injection of chemically misacylated tRNA (e.g., BODIPY-FL^205^ or Cy dyes^206^). In these cases, low encoding efficiency has been observed, limiting application to single molecule methods such as TIRF, and thus far failing to achieve significant biological or mechanistic insight.

The applications of and requirements for fluorescence approaches lend themselves especially well to scalable recombinant systems. In this regard, a few orthogonal tRNA/synthetase pairs specific for environmentally sensitive (ANAP and Coumarin^207−209^) and photostable (Acd^210,211^) fluorophores have been identified thus far (Figure 7B) . Of these, the ANAP^209^ fluorophore system has proven especially versatile and sufficiently robust for macroscopic fluorescence techniques. In channels, the synthetase/tRNA pair was pioneered in the encoding of ANAP into the *Shaker* K_V_ expressed in *Xenopus* oocytes.^212,213^ These *Shaker* studies have demonstrated a clear benefit of genetically encoded fluorescent noncanonical amino acids: the freedom to install probes at internal ion channel positions that are not accessible to aqueous thiol modification.^212,213^ Similar studies observing channel state- dependent changes in fluorescence (intensity and/or spectra) have been performed in Na_v_,^214^ ASIC,^215^ and GlyR^216^ channels.

The Gordon and Zagotta groups extended the use of ANAP as a FRET donor^217^ in channels expressed in mammalian cells to measure state-dependent distances with nonfluorescent transition metal acceptors on the plasma membrane or on the channel itself.^218−221^ ANAP was also used as a FRET donor in the nucleotide binding domain of K_ATP_ channels, with fluorescent nucleotides as acceptors.^222^ Recently, ANAP was encoded at multiple positions on H_v_ channels to study the voltage and pH dependence of domain movement.^223^ Interestingly for this VCF study, for one position of ANAP encoding (A197) on the S4 voltage sensing helix, an observed increase in fluorescence intensity upon depolarization was evidently not chiefly due to changes in environment hydrophobicity but from movement away from a quenching Phe residue on the neighboring S2 helix (F150). This general mechanism has precedent in other fluorophore-aromatic pairs (e.g., bimane quenching by tryptophan^224^ and tyrosine^225^), and the finding suggests that ANAP (as a single label) may be used to study trajectories and relative distances in the right structural contexts.

Overall, given the extent to which the ion channel field has extensively utilized ANAP for novel structure–function insights, an exciting area of opportunity for major technical advancement is the future identification of novel synthetases specific for fluorophores with a wide range of spectral characteristics. Parallel and complementary advancements in the encoding of noncanonical amino acids for orthogonal “click” chemistries are also expected to enable increases in specificity for fluorophores,^226,230^ and other spectroscopic probes,^227^ over traditional labeling methods.

## 6 Conclusions and Outlook

The mechanistic bases for ion channel function and pharmacology have been substantially elevated through the adoption and application of GCE methods for the encoding of noncanonical amino acids. These efforts have produced high resolution details of structure–function relationships and ligand–receptor binding details that would have been otherwise unknowable. These successes are, in no small part, due to the exquisite sensitivity of measurements that can be used to assess channel function and access to validated, rigorous biophysical methods for their study. In turn, the explosion in atomic and near-atomic resolution structures of ion channels has greatly facilitated the interpretation of GCE results and revealed new functional centers to interrogate. Going forward, new fluorescent probes have the potential to illuminate the functional details of protein conformational changes in ion channels and other proteins. It is expected that ongoing improvements in system efficiency and scalability as well as breakthroughs in terms of new chemistries to be encoded will open new experimental theaters. The continued democratization of reagents and methods will be at the forefront of these developments as the GCE ecosystem expands. Accessibility is enhanced by many diverse means. Most obviously, technical advancements can enable greater adoption through methodological simplification. For example, for the chemical misacylation approach for nonsense suppression, chemical deprotection of acylated tRNA enables simple resuspension and injection, without the need for sensitive UV deprotection, thus enabling the sharing of these reagents between laboratories. While this approach involves significant knowledge of synthetic chemistry, our group has formed an NIH funded resource for the distribution of such reagents.^228^ Additionally, for some ncAAs traditionally encodable only through the (chemistry-heavy) tRNA misacylation method, specific tRNA/synthetase pairs are now available (e.g., fluorinated phenylalanine^115^ and photocaged histidine^66^). Finally, collaborative resource sharing and training^229^ is essential for this progress to continue at pace.
